# Prelinguistic human infants and great apes show different communicative strategies in a triadic request situation

**DOI:** 10.1371/journal.pone.0175227

**Published:** 2017-04-06

**Authors:** Heinz Gretscher, Sebastian Tempelmann, Daniel B. M. Haun, Katja Liebal, Juliane Kaminski

**Affiliations:** 1 Department of Developmental and Comparative Psychology, Max Planck Institute for Evolutionary Anthropology, Leipzig, Germany; 2 University of Applied Sciences and Arts Northwestern Switzerland, Liestal, Switzerland; 3 Cluster Languages of Emotion, Freie Universität Berlin, Berlin, Germany; 4 Leipzig Research Center for Early Child Development, Leipzig University, Leipzig, Germany; 5 Department of Early Child Development and Culture, Leipzig University, Leipzig, Germany; 6 Centre for Comparative and Evolutionary Psychology, University of Portsmouth, Portsmouth, United Kingdom; National Institute of Child Health and Human Development, UNITED STATES

## Abstract

In the present research, we investigate the communicative strategies of 20 month old human infants and great apes when requesting rewards from a human experimenter. Infants and apes both adapted their signals to the attentional state of the experimenter as well as to the location of the reward. Yet, while infants frequently positioned themselves in front of the experimenter and pointed towards a distant reward, apes either remained in the experimenter’s line of sight and pointed towards him or moved out of sight and pointed towards the reward. Further, when pointing towards a reward that was placed at a distance from the experimenter, only the infants, and not the apes, took the experimenter’s attentional state into account. These results demonstrate that prelinguistic human infants and nonhuman apes use different means when guiding others’ attention to a location; indicating that differing cognitive mechanisms may underlie their pointing gestures.

## Introduction

While two month old human infants readily engage in dyadic face-to-face interactions, it is not until the age of 9–12 months before they start to communicate about objects and events. This inclusion of external entities into their interactions with others is first achieved through triadic gestures. Triadic gestures are gestures that reference external entities such as objects or events, for instance by showing, offering or pointing [1].

Bates et al. [1] classified these early referential gestures as either (proto-)imperative or (proto-)declarative. According to them, infants’ imperative gesturing constitutes a kind of social tool use in which others are used as a means to obtain a desired object. Declarative gestures, on the other hand, are used by the gesturing individual to direct the recipient’s attention to the referent. This has led some to hypothesize that declarative gestures indicate an understanding of unobservable mental states, whereas employing imperative gestures merely requires an understanding of others as causal agents (e.g. [2, 3, 4]).

Paralleling this distinction between imperative and declarative gestures, there has been a long-running debate whether infant prelinguistic communication is cognitively ‘lean’ or ‘rich’ (reviewed in [5]). According to a cognitively lean interpretation of infant communication infants initially gesture for selfish reasons and without any deeper understanding of the mental states of others [6, 7]. Declarative gesturing and other joint attention behaviours are thought to develop by reinforcement through external rewards such as attaining interesting objects or the adults’ attention [8]. Empirical support comes mainly from two lines of evidence. First, imperative pointing seems to emerge before declarative pointing [9]. Second, infants with autism and infants with Down syndrome, who are known to suffer from socio-cognitive deficits, do not point declaratively but still engage in imperative pointing [2, 10].

A rich interpretation of infant communication assumes that from the onset, infants’ early gestural communication goes beyond the achievement of material goals and involves an understanding of others as mental agents, with their referential intentions aimed at altering and directing the mental states of others (reviewed in [11]). In this view, the simultaneous appearance of triadic gesturing and other joint attention behaviors indicates a common underlying developmental process—namely the infants’ developing understanding of others as mental agents like themselves [12]. Indeed, when human infants start to point at around 12 months of age, they follow the gaze of others to out of sight targets [13], around visual barriers [14], check back with the looker if they do not encounter a potential gaze target [15] and (around 14 months) understand the role of eyes in vision as well as the occluding properties of blindfolds [16].

Overall, these findings suggest that infants understand the gazing of others as ‘seeing’. However, much less is known about infants’ understanding of others’ auditory perception. Moll et al. [17] found that 2-year-olds know what others have heard in the immediate past based on previous episodes of joint auditory attention. Two-year-olds in a study of Williamson et al. [18] produced higher intensity sounds when prompted to wake a doll whereas they produced lower intensity sounds after being told not to wake the doll. In a study of Melis et al. [19], 3-year-olds, in the presence of an adult, preferentially retrieved a prohibited toy through a ‘silent door’ instead of a ‘noisy door’ (which had a bell attached to it). However, to our knowledge, no study so far directly investigated whether infants employ auditory gestures or vocalizations to attract attention in a communicative context.

For nonhuman great apes (henceforth apes), it has also been debated whether they gesture to influence others’ mental states, or alternatively, to simply influence others’ behaviour. Apes flexibly use a particular gesture across different functional contexts and a variety of gestures to achieve the same end in a specific context [20]. When pursuing a communicative goal, they show persistence as well as elaboration of their gestures (e.g. [21]). Further, apes understand that recipients have to be perceptually attentive for a signal to be effective and adapt their visual signals accordingly (e.g. [22–24]). The evidence that apes use auditory signals as attention getters is mixed, although Melis et al. [25] found that chimpanzees preferentially retrieved a contested piece of food through a silent door instead of a noisy one if a human competitor was present, suggesting that they might be aware of others’ auditory perception capacities (but see [26]). Similarly, apes have been evidenced to respond to an inattentive recipient with an increase in auditory response measures [24,27–29]. However, other studies have found no effects of the recipient’s orientation on auditory signaling [23,30] or a decrease of auditory signals in response to an inattentive recipient [31,32]. While apes demonstrate a generally high degree of flexibility in their gestural communication, their gesturing appears to mostly serve imperative purposes. This also includes ape manual pointing (henceforth ‘pointing’).

Whereas pointing is only rarely observed in the wild (but see [33]), captive apes sometimes spontaneously acquire the pointing gesture (i.e., without any explicit training). However, most apes exclusively point for human caregivers, but only rarely for other conspecifics (reviewed in [34]). Further, ape pointing usually occurs either as a direct imperative request for food or as indirect imperative request for tools that are instrumental in retrieving food (e.g. [35, 36]) with the exception of home reared and language-trained apes who have also been reported to engage in declarative pointing (reviewed in [37]); albeit that imperative requests apparently constitute the major part of their communication [38]. Moreover, standard-reared captive apes’ failure to comprehend pointing cues in the object-choice task, in which a human experimenter points to the one container out of several possible that is holding hidden food, casts doubt on a cognitive rich interpretation of ape pointing (see [39] also for contradictory results with language-trained apes). Nevertheless, the high level of sophistication that apes show in their gestural communication has led some researchers to claim that “pointing, per se, does not require cognitive adaptations that are unique to humans” ([34], p. 86); that apes gesture referentially, and that these gestures can be considered triadic (e.g., indicated by gesturing for the most desirable food; [40]). Thus, in such a triadic request situation it remains ambiguous whether the apes only point to receive the food or (also) to direct the recipient’s attention.

Although developmental and comparative psychologists tend to classify pointing and other gestures as either imperative or declarative, some studies show that this distinction might be artificial and that infants’ imperative requests sometimes include an attempt to influence others’ mental states [41,42]. Therefore, as indicated by Halina [43], from a cognitive perspective it might be more fruitful to characterize pointing (and other gestures) according to whether or not it constitutes a deliberate attempt to direct the gaze of others (to an external entity). However, if the external entity is constantly in the attentional focus of the recipient, as is the case in most studies on pointing production with apes where the location of the food and the recipient is identical, there is no need to direct attention via gestures. Thus, in such a triadic request situation it remains ambiguous whether the apes only point to receive the food or (also) to direct the recipient’s attention.

One notable exception where the recipient was at a separate location than the referent is the study of Roberts et al. [44] in which two language-trained chimpanzees used their gestures to guide a human helper to distant hidden food. Whereas both chimpanzees increased the frequency of their gesturing when the human approached the food, only one of the two actually pointed in the direction of the food. Yet, due to the repeated turn-taking and communicative interactions with the human, it remains unclear whether the apes were attempting to direct the human’s attention towards the food (or were just responding to behavioural and positional cues to maneuver the human to the reward location). Furthermore the results of the only other study investigating apes’ communication in a situation in which food and experimenter were at separate locations [45] are difficult to compare to other studies (e.g. [46]). This is due to the lack of control conditions examining apes’ performance (1) in a situation in which the experimenter and the food were at the same location or (2) in a situation in which the experimenter was inattentive. Further, whereas plenty of studies have established that infants point to direct (and share) attention, only a minority has investigated whether this also holds true for infants’ gestural requests (but see [41,42]).

Thus, to explore the communicative strategies of prelinguistic human infants and great apes in a comparable triadic request situation we systematically varied the location of the referent (i.e., the reward) relative to the experimenter (the reward and the experimenter either being separated by an opaque barrier or not), as well as the experimenter’s visual attention (the experimenter either facing towards the participant or not) in a two by two factorial design. We expected participants to adapt their signals to the attentional state of the experimenter (e.g., by producing less visual gestures and more auditory signals when the experimenter was facing away). If some of the participants’ requests constituted a deliberate attempt to direct the experimenter’s attention to the reward, we were expecting participants to employ those communicative signals in such ways that they were not only visible to the experimenter, but that they were also specifically targeted towards the reward. Thus, when the experimenter was separated from the reward, to qualify as triadic the respective signals should (1) be targeted towards the reward (instead of towards the experimenter) and (2) be directed to the experimenter (i.e., be produced on the experimenter’s side of the barrier and be more frequent when the experimenter was turned towards the participant than when he was turned away). Participants’ performance in the conditions in which experimenter and the reward were located on the same side will help to contrast the results with unambiguously triadic conditions (i.e., when the reward was placed separately from the experimenter) to the results of other studies. As Menzel [47] suggested inconspicuous body pointing to be a possible mechanism for referential communication in chimpanzees, we analysed participants’ movement between sides (‘switches’) in addition to their gestural and auditory signaling.

## Methods

### Participants

Forty-one human infants were recruited through the child laboratory of the Max Planck Institute for Evolutionary Anthropology in Leipzig, Germany. Seventeen infants had to be excluded: 14 of them lost interest in the toy apparatus that served as motivational incentive for requesting reward before completing one session of all four experimental conditions, and three due to experimenter error. The final dataset consisted of a total of 12 boys and 12 girls with a mean age of 19.94 months (*SD* = 0.30 months; range: 19.48–20.47 months). The infants were tested individually in the presence of a parent in a silent room at the institute, in which they were allowed to move freely (i.e., infants were not restrained by being seated on the parent’s lap or on a chair). Parents were instructed to refrain from any verbal or non-verbal cuing. Informed written consent was obtained from all the parents of the infants who participated in this study. The study was non-invasive and adhered to all appropriate German ethical and legal protocols. Furthermore, the study procedure was approved by the Ethics Committee of the Max Planck Institute for Evolutionary Anthropology Ethics Committee.

Thirty-two apes, housed at the Wolfgang Köhler Primate Research Centre in Leipzig, Germany, participated in the study. Participants were five bonobos (*Pan paniscus*, 2 females, 3 males), 18 chimpanzees (*Pan troglodytes*, 13 females, 5 males), three gorillas (*Gorilla gorilla*, 3 females) and six orangutans (*Pongo pygmaeus*, 5 females, 1 male). Participants’ mean age was 17.8 years (*SD* = 9.7 years; range: 4.4–34.9 years). Groups of apes were housed in semi-natural indoor and outdoor enclosures with regular feedings, daily enrichment and water was available ad libitum. Participants voluntarily participated in the study and were tested individually (or with their dependent offspring) in familiar sleeping or observation rooms. Research at the WKPRC was performed in accordance with the recommendations of the Weatherall report “The use of nonhuman primates in research” [48] and strictly adhered to the legal requirements of Germany. No medical, toxicological or neurobiological research of any kind is conducted at the WKPRC. The full procedure of the study was approved by the Max Planck Institute for Evolutionary Anthropology Ethics Committee. Animal husbandry and research comply with the “EAZA Minimum Standards for the Accommodation and Care of Animals in Zoos and Aquaria”, the “WAZA Ethical Guidelines for the Conduct of Research on Animals by Zoos and Aquariums” and the “Guidelines for the Treatment of Animals in Behavioral Research and Teaching” of the Association for the Study of Animal Behavior (ASAB). IRB approval was not necessary because no special permission for the use of animals in purely behavioral or observational studies is required in Germany. Further information on this legislature can be found in paragraphs 7.1, 7.2 and 8.1 of the German Protection of Animals Act (“Tierschutzgesetz”).

### Experimental set-up

**Fig 1:**
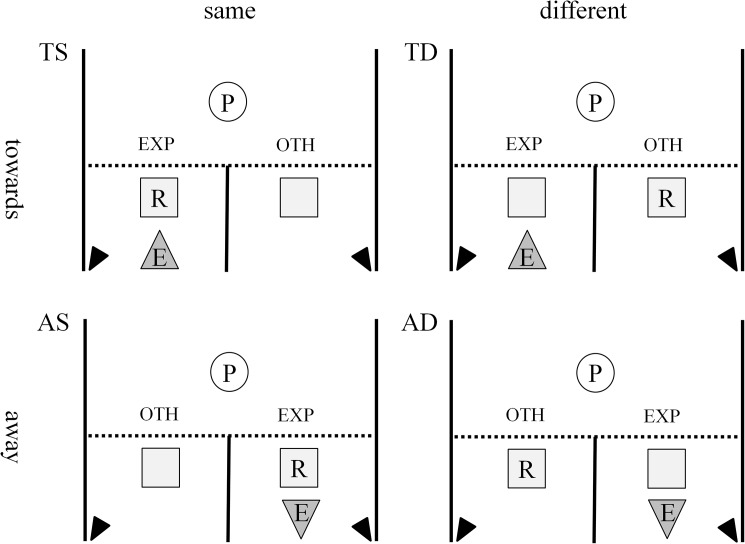
Experimental set-up in all conditions. (R = reward; E = experimenter; P = participant; dotted line = transparent barrier; central solid line = opaque visual barrier; squares = containers; black triangles at the bottom left and right corners: cameras). In a two by two study design we varied whether the experimenter was oriented towards the participant (upper row; indicated by a triangle facing upwards) or away from the participant (lower row; indicated by a triangle facing downwards) and whether the experimenter and the reward were located on the same side (left column) or on different sides (right column) resulting in the four experimental conditions towards-same (TS), towards-different (TD), away-same (AS) and away-different (AD); EXP: experimenter side, OTH: other side.

Two containers were used (approximately 1.5 m apart), one of them holding a reward and the other one remaining empty (see Fig 1). Participant and experimenter (*E1*) were separated by a transparent barrier allowing participants to see both containers but not to reach them. The experimenter’s area was split in half by another, visually opaque, barrier, blocking the line of sight between the experimenter’s side and the other side.

Infants were rewarded with coloured wooden blocks that they could put down a chute to elicit sounds from a toy apparatus held by a second experimenter (*E2*) at the far end of the room. Both *E1* and *E2* were unfamiliar to all infants. Two cardboard boxes were used as containers to stash the blocks. As pilot testing revealed that infants had difficulties to remember where the reward was hidden, the boxes were left open and placed with the top opening facing the participant. Thus, the infant (but not *E1*) was able to see the blocks in warm-up as well as experimental trials. The transparent plexiglass barrier was 80 cm high with a narrow gap in the midst of each side to facilitate the transfer of blocks between experimenter and infant. Before entering the testing room the infants became acquainted with both experimenters in a short warm-up play (of about 10 min duration) with various toys unrelated to the study.

Apes received food as reward. Opaque plastic cups were used as containers to hide food pieces. The transparent plexiglass barrier had two small holes on each side allowing the apes to indicate their choice of cups. *E1* and *E2* were familiar to all apes because of previous studies.

### Warm-up trials

Prior to testing, all participants received a series of warm-up trials to accustom themselves to the general setup and procedure of the task. Participants were familiarized with requesting a reward from the experimenter as well as the fact that rewards could be obtained on both sides of the barrier.

The infants’ warm-up started with *E1*, *E2*, and the infant conjointly exploring the toy apparatus. After a brief introduction into its functionality, *E1* entered the experimenter’s area and sat down on the right side of the opaque barrier (from *E1*’s perspective) oriented towards the infant. *E1* responded to the infant’s gestural requests by handing over blocks (one at a time) until the container was empty. The same procedure was repeated on the left side of the opaque barrier. In total, infants received six blocks from *E1* (three on each side). If infants did not spontaneously request blocks, *E2* verbally encouraged them to do so, occasionally also guiding them to *E1* and the container.

Ape warm-up trials started with *E2* placing a piece of food under one of two cups (both positioned either on the right side or the left side of the opaque barrier) in full sight of the participant. After *E2* had left the room, *E1* entered and sat down in front of the cups, with his face and body turned towards the participant. Participants had only one chance to indicate their choice of cups. They only received the food reward if they chose the baited cup. Subsequently, *E1* left and *E2* re-entered to set up the next trial. Participants received a total of six warm-up trials (three on the left side and three on the right side of the opaque barrier). In half of the trials the reward was placed under the right cup, in half of the trials it was placed under the left cup. Warm-up trials were semi-randomized such that no more than two consecutive trials were conducted on the same side. Likewise, neither could the reward be retrieved from the same cup in more than two consecutive trials.

### Experimental trials

In the experimental trials we systematically varied the two factors of (whole body) orientation and location (in regards to the reward) in a two-by-two study design. *E1* could be oriented either *towards* the participant or *away* from the participant and *E1* and the reward could be either located on the *same* side or on *different* sides of the visual barrier (see Fig 1). This resulted in the following four experimental conditions.

1. ***towards-same (TS)*:** *E1* was facing the participant and *E1* and the reward were both located on the experimenter’s side (see S1 Video).
2. ***towards-different (TD)*:** *E1* was facing the participant and located on the experimenter’s side whereas the reward was located on the other side (see S2 Video).
3. ***away-same (AS)*:** *E1* was oriented 180 degrees away from the participant and *E1* and the reward were both located on the experimenter’s side (see S3 Video).
4. ***away-different (AD)*:** *E1* was oriented 180 degrees away from the participant and located on the experimenter’s side whereas the reward was located on the other side (see S4 Video).

During trials, *E1* maintained the initially assumed posture and position, acted neutrally (i.e., by performing slight movements or producing humming sounds to make the situation more natural) and never responded to any of the participant’s behaviour. Furthermore, *E1* avoided direct eye contact with the participant during testing, as eye contact itself is known to be a strong communicative cue [49]. After the trial duration had elapsed, *E1* revealed the content of the container that the participants showed the strongest interest in (indicated by their position and communicative behaviour). Participants received non-differential reinforcement, that is, *E1* always retrieved and transferred the reward from the baited container, either directly (when participants chose correctly) or after first revealing the empty container (when participants chose incorrectly). *E1* then left the scenery while *E2* set up the next trial. All participants completed at least one session, each consisting of four trials (one per condition). The order of conditions within a session was counterbalanced across participants.

For infants eight trials were administered, split up in two consecutive sessions on the same day. Trial duration was 20 s. While 12 out of 24 infants completed both sessions, the others completed only the first session and the second session partially (average number of trials completed = 6.8; *SD* = 1.4).

For apes, 16 trials were administered, each lasting 30 s, on two separate days with two consecutive sessions on each day. On both days, apes received a warm-up prior to testing and two additional motivational trials in-between the two sessions. Motivational trials were identical to warm-up trials, with one of them being administered at each side.

### Coding

All trials were recorded by digital camcorders from different angles (one camera recording on each side for infants, two per side for apes: one recording at the ground level, one recording at the upper ceiling level of the testing area) and subsequently scored from the video files by the first author (infants) and a second person (apes). We coded a total of 15 different communicative behaviours (see Table 1), the side where participants were located at when producing the behaviour (‘side of production’) and towards which ‘target side’ it was directed. The three possible values for ‘Side’ were ‘experimenter’s side’ (i.e., the side where *E1* was located), ‘other side’ (i.e., the side that *E1* could not see), and ‘middle’ (i.e. the area in-between sides; a participant was scored as being in the middle position when its body parts were spanning the experimenter’s side as well as the other side). To exclude interactions with other communicative partners than the experimenter (e.g., with a parent or *E2*), a behaviour was only scored if the participant was oriented towards the experimenter’s area (i.e., experimenter’s side, other side or the middle). For analysis, we categorized the 15 different behaviours according to their main mode of perception: auditory signals and visual gestures. As Menzel [49] suggested inconspicuous body pointing to be a possible form of referential communication in chimpanzees, we also scored two positional measurements. These were movements from one side to the other (‘switches’), and the time each participant spent on either side (‘duration of stay’). A second coder, blind to the experimental condition, coded the participants’ behaviour in 18% of all conducted trials. Agreement between coders was 85% for behaviour types (Cohen’s κ = .77, *N* = 616), 96% for sides of production (κ = .92, *N* = 432), and 98% for target sides towards that apes directed their gestures (κ = .97, *N* = 359). Intraclass correlation for the duration of stay was ICC(1,1) = .92.

**Table 1:** Definition of communicative behaviours classified according to their respective sensory modality.

Signal type	Infants	Apes	Definition
Visual			
Offer		17 (8)	Transfer of an object through the transparent barrier
Empty hands	5 (1)		Presenting one or both hands with arms bent and palms up
Point	77 (18)	1442 (32)	Goal directed extension of one (or more) finger(s) with an accompanying arm movement (including insertion of fingers through the holes in the transparent barrier)
Raise arm	1 (1)		Upwards lifting of one (or both) arm(s)
Reach	29 (11)		Goal directed arm movement with an open hand (without finger extension)
Request		27 (13)	Pressing the mouth against or cupping a hand before a hole of the transparent barrier
Shake hand		38 (7)	Repeated shaking of one (or both) hand(s)
Shake head	2 (2)	18 (5)	Repeated moving of the head from side to side or up and down
Shrug	2 (2)		Rapid lifting or contraction of the shoulders
Auditory			
Body slap	3 (2)	2 (2)	Noisily hitting one’s own body
Clap		10 (3)	Slapping hands or feet together
Jump		3 (2)	Jumping with both feet of the ground
Bang	29 (9)	127 (23)	hitting, kicking, knocking, pushing or shaking an item (barrier, wall or ground) such that a noise is produced
Spit		7 (3)	Spitting through the panel
Vocalization	256 (21)	17 (5)	Production of vocal sounds

Note. Summarizing all sessions, the columns ‘infants’ and ‘apes’ show the total number of occurrences (first number) and the number of individuals that used the respective signal (second number, in brackets).

### Analysis

One of the goals of the study was to investigate whether apes and infants exhibit a preference to signal in close proximity to the reward [46] or whether they show no such preference [45]. Therefore we explored for each condition whether participants spent more time or produced more auditory and visual gestures on the experimenter’s side or on the other side. Wilcoxon signed-rank tests were employed to conduct within condition across side comparisons for each of the three aforementioned behavioural measurements.

Further, the variation of the number of side switches as well as auditory and visual signals and pointing gestures was analysed separately for each side. For auditory signals and visual gestures we analysed the *sides of production* (i.e., where participants were located during their request); for side switches and pointing, we analysed *target sides* (i.e., towards which sides participants directed these behaviours). If possible, we assessed the overall and main effects of the three predictor variables species (human vs. ape), orientation (towards vs. away), and location (experimenter’s side vs. other side) as well as their interaction via a Generalized Linear Mixed Model (GLMM). If a GLMM indicated a significant interaction between the test factors orientation and location, follow-up pairwise comparisons via Wilcoxon signed-ranks tests were conducted across each one of the factors while the other was held constant. As we were primarily interested in whether the test factors orientation and location differentially affected infants’ and apes’ behaviours and less in absolute differences between species (which also might have been due to differences in the experimental procedures), post hoc analyses for interactions involving the factor species were only conducted within and not across species. As the data for the number of pointing gestures directed towards the other side was too sparse to run a GLMM analysis (leading to a convergence failure within 100,000 iterations), we used Wilcoxon signed-ranks tests to compare the factors orientation and location separately for each species.

GLMM full models included the three main factors species, orientation and location, their interaction, the fixed control variables sex and (z-transformed) trial order and, to control for random by-participant variation, the random effect as well as the random slopes of participants. All analyses were conducted using the statistic software R [50]. The count data was modeled with a Poisson distribution via the lme4-package [51]. To test the overall significance of the two test factors, likelihood ratio tests (based on the -2LL values of the respective models) were used to compare the full model to a null model containing only the control variables and random slopes [52]. All full models that differed significantly from their respective null models were compared to a reduced model that did not include the three-way interaction of the test factors but was otherwise identical to the corresponding full model. Subsequently the reduced model containing all two-way interactions was compared to reduced models that lacked one of the three two-way interactions (but were otherwise identical). Non-significant interactions were removed from the model to interpret lower-order effects. Throughout the results section by referring to ‘participants’ we denote the combined datasets of apes and infants.

The ape sample sizes (with all species except for chimpanzees being composed by as few as six or less individuals) prevented us from including species as a five level categorical factor in the GLMMs and conducting (meaningful) post hoc comparisons separately for each ape species. However, we additionally ran all analyses with a reduced ape sample restricted to the two *Pan* species (see S1–S7 Tables). If not stated otherwise these analyses did not yield differing results from the analyses combining all nonhuman genera as ‘apes’.

## Results

First, we will present the analysis of the two positional measurements ‘duration of stay’ and ‘side switches’. We will then focus on the production of auditory and visual signals, and conclude the results section by analyzing to which target sides participants directed their pointing gestures.

### Duration of stay

For infants, across side comparisons revealed that they stayed significantly longer on the experimenter’s side if the reward was located on the same side (Wilcoxon signed-ranks test; TS: *T*_*+*_ = 254.0, *N* = 23, *p* < .001; AS: *T*_*+*_ = 214.0, *N* = 22, *p* = .003). When the experimenter and the reward were located on different sides, infants showed no preference for either side (TD: *T*_*+*_ = 145.0, *N* = 21, *p* = .320; AD: *T*_*+*_ = 119.5, *N* = 20, *p* = .601). Apes stayed significantly longer on the experimenter’s side if the reward was located on the same side (TS and AS: *T*_*+*_ = 528.0, *N* = 32, *p* < .001). If the reward and the experimenter were located on different sides, apes preferred to stay on the other side, that is, with the reward (TD: *T*_*+*_ = 413.0, *N* = 32, *p* = .004; AD: *T*_*+*_ = 450.0, *N* = 31, *p* < .001).

### Switches

**Fig 2:**
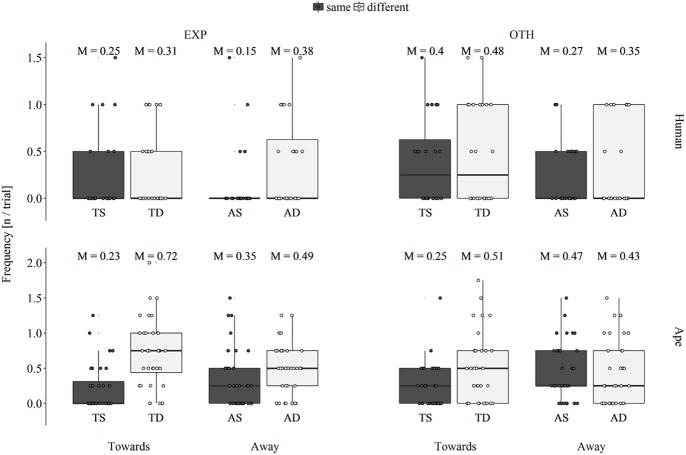
Box plot of the average number of times per trial that human infants (upper row) and apes (lower row) switched to the experimenter’s side (EXP: left column) and the other side (OTH: right column) across the factors orientation (Towards: two leftmost boxes in each plot, Away: two rightmost boxes in each plot) and location (red: experimenter and reward are on the same side, blue: experimenter and reward are on different sides) resulting in the four experimental conditions towards-same (TS), towards-different (TD), away-same (AS) and away-different (AD); M: condition mean value, the middle line of each box represent population median values (different from the M above each box); lower and upper hinges display first and third quartiles, respectively; whiskers extend up to 1.5 x interquartile range, small circles represent individual data points.

A Poisson GLMM indicated a significant overall effect of the three test factors on the number of switches to the experimenter’s side (likelihood ratio test: χ^2^ = 48.16, *df* = 7, *p* < .001; see Fig 2). This was due to a significant interaction of orientation and location (χ^2^ = 5.43, *df* = 1, *p* = .020; see S1 Table) and apes switching to the experimenter’s side more often than infants (main effect of species: 0.53 ± 0.26, *p* = .038). Post hoc pairwise comparisons within orientation revealed that participants switched more often to the experimenter’s side when the experimenter and the reward were located on different sides (Wilcoxon signed-ranks test; TS–TD: *T*_*+*_ = 755.0, *N* = 43, *p* < .001; AS–AD: *T*_*+*_ = 414.0, *N* = 32, *p* = .003). Comparisons within location indicated no significant differences across orientation (TS–AS: *T*_*+*_ = 172.5, *N* = 24, *p* = .526; TD–AD: *T*_*+*_ = 456.0, *N* = 37, *p* = .114).

A second Poisson GLMM also indicated a significant overall effect of the three test factors on the number of switches to the other side (likelihood ratio test: χ^2^ = 15.93, *df* = 7, *p* = .026), due to a significant interaction of orientation and location (χ^2^ = 6.19, *df* = 1, *p* = .013; see S2 Table). Post hoc pairwise comparisons across location revealed that within the two *towards* conditions, participants switched more often to the experimenter’s side when the experimenter and the reward were located on different sides (Wilcoxon signed-ranks test; TS–TD: *T*_*+*_ = 535.0, *N* = 38, *p* = .015), whereas there was no such difference when the experimenter was inattentive (AS–AD: *T*_*+*_ = 238.5, *N* = 30, *p* = .896). Comparisons within location indicated no significant differences across orientation (TS–AS: *T*_*+*_ = 320.0, *N* = 31, *p* = .178; TD–AD: *T*_*+*_ = 317.0, *N* = 31, *p* = .178).

### Auditory signals (including vocalizations)

Across sides, infants produced significantly more auditory signals on the experimenter’s side in three out of four conditions (TS: *T*_*+*_ = 91.0, *N* = 13, *p* < .001; TD: *T*_*+*_ = 129.5, *N* = 17, *p* = .010; AS: *T*_*+*_ = 93.0, *N* = 14, *p* = .008). In the AD condition, infants showed no preference for either side (*T*_*+*_ = 78.5, *N* = 15, *p* = .307). Apes produced more auditory signals at the experimenter’s side when the reward was on the same side (TS: *T*_*+*_ = 231.0, *N* = 21, *p* < .001; AS: *T*_*+*_ = 160.0, *N* = 18, *p* < .001), whereas they showed no preference for either side in the TD condition (*T*_*+*_ = 20.5, *N* = 8, *p* = .805) and produced more auditory signals on the other side in the AD condition (*T*_*+*_ = 58.5, *N* = 11, *p* = .020). The latter difference resulted only in a trend when just the two *Pan* species were considered (*T*_*+*_ = 31.0, *N* = 8, *p* = .078).

A Poisson GLMM analysis indicated a significant overall effect of the test factors on the number of auditory signals that participants produced on the experimenter’s side (likelihood ratio test; χ^2^ = 53.45, *df* = 7, *p* < .001). This was due to a significant interaction of species and location (χ^2^ = 13.84, *df* = 1, *p* < .001; see S3 Table) and a trend to produce more auditory signals when the experimenter was oriented towards the participants (main effect of orientation: 0.34 ± 0.20, *p* = .098). When only the two *Pan* species were included, latter difference resulted in a significant effect (main effect of orientation: 0.45 ± 0.20, *p* = .029). Post hoc pairwise comparisons across location revealed that apes produced more auditory signals on the experimenter’s side when the reward was located on the same side (Wilcoxon signed-ranks test; *T*_*+*_ = 253.0, *N* = 22, *p* < .001), whereas no such difference was found for infants (*T*_*+*_ = 132.0, *N* = 21, *p* = .579).

A second Poisson GLMM analysis indicated also a significant overall effect of the test factors on the number of auditory signals that participants produced on the other side (likelihood ratio test; χ^2^ = 64.68, *df* = 7, *p* < .001). This was due to a interaction of species and orientation (χ^2^ = 3.89, *df* = 1, *p* = .048; see S4 Table) that turned to a trend when the ape sample was restricted to include only the two *Pan* species (χ^2^ = 2.51, *df* = 1, *p* = .078), and participants producing more auditory signals on the other side when the experimenter and the reward were located on different sides (main effect of location: -4.09 ± 1.95, *p* = .036). Post hoc pairwise comparisons indicated that neither infants nor apes differed in the number of auditory signals produced on the other side across orientation (Wilcoxon signed-ranks test; infants: *T*_*+*_ = 40.0, *N* = 10, *p* = .229; apes: *T*_*+*_ = 75.5, *N* = 14, *p* = .169).

### Visual gestures

**Fig 3:**
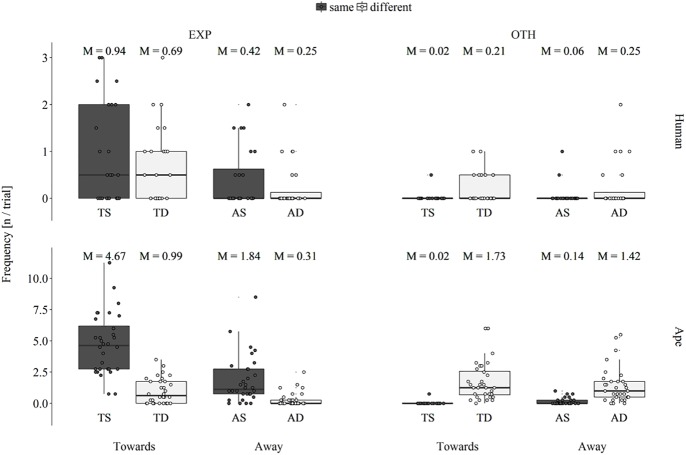
Box plot of the average number of visual gestures per trial that human infants (upper row) and apes (lower row) produced on the experimenter’s side (EXP: left column) and the other side (OTH: right column) across the factors orientation (Towards: two leftmost boxes in each plot, Away: two rightmost boxes in each plot) and location (red: experimenter and reward are on the same side, blue: experimenter and reward are on different sides) resulting in the four experimental conditions towards-same (TS), towards-different (TD), away-same (AS) and away-different (AD); M: condition mean value, the middle line of each box represent population median values (different from the M above each box); lower and upper hinges display first and third quartiles, respectively; whiskers extend up to 1.5 x interquartile range, small circles represent individual data points.

Across sides, infants produced a higher number of visual gestures at the experimenter’s side in three out of four conditions (TS: *T*_*+*_ = 105.0, *N* = 14, *p* < .001; TD: *T*_*+*_ = 53.0, *N* = 10, *p* = .008; AS: *T*_*+*_ = 41.0, *N* = 9, *p* = .027; see Fig 3). In the AD condition they showed no preference for either side (*T*_*+*_ = 35.0, *N* = 11, *p* > .922). Apes exhibited a preference to produce visual gestures in proximity to the reward, that is, they visually gestured more frequently on the experimenter’s side when the reward was located on the same side (TS: *T*_*+*_ = 528.0, *N* = 32, *p* < .001; AS: *T*_*+*_ = 458.0, *N* = 30, *p* < .001), whereas they produced more visual gestures on the other side when the experimenter and the reward were located on different sides (TD: *T*_*+*_ = 363.0, *N* = 31, *p* = .023; AD: *T*_*+*_ = 383.0, *N* = 28, *p* < .001). If the analyses were restricted to the two *Pan* species, the difference across sides in the TD condition turned into a trend (TD: *T*_*+*_ = 193.5, *N* = 23, *p* = .093).

A Poisson GLMM indicated a significant overall effect of the test factors on the number of visual gestures that participants produced on the experimenter’s side (likelihood ratio test; χ^2^ = 103.85, *df* = 7, *p* < .001). This was due to a significant interaction of species and location (χ^2^ = 20.39, *df* = 1, *p* < .001; see S5 Table) and participants visually gesturing more frequently when the experimenter was oriented towards them (main effect of orientation: 1.07 ± 0.13, *p* < .001). Post hoc pairwise comparisons indicated that apes produced more visual gestures on the experimenter’s side when the reward was located on the same side (Wilcoxon signed-ranks test; *T*_*+*_ = 496.0, *N* = 31, *p* < .001), whereas no such difference was found for infants (*T*_*+*_ = 114.0, *N* = 18, *p* = .223).

A second Poisson GLMM revealed that the test factors also had a significant overall effect on the number of visual gestures that participants produced on the other side (likelihood ratio test; χ^2^ = 124.97, *df* = 7, *p* < .001), due to a significant interaction of orientation and location (χ^2^ = 14.41, *df* = 1, *p* < .001; see S6 Table) and apes producing more visual gestures than infants (main effect of species: 1.83 ± 0.35, *p* < .001). Post hoc pairwise comparisons within orientation revealed that participants produced more visual gestures on the other side when the experimenter and the reward were located on different sides (TS–TD: *T*_*+*_ = 741.0, *N* = 38, *p* < .001; AS–AD: *T*_*+*_ = 603.5, *N* = 35, *p* < .001). Comparisons across orientation indicated that participants within the two same conditions produced more visual gestures on the other side when the experimenter was oriented away (Wilcoxon signed-ranks test; TS–AS: *T*_*+*_ = 66.0, *N* = 12, *p* = .033). Contrary, pairwise comparisons within the two different conditions indicated no significant differences across orientation (TD–AD: *T*_*+*_ = 370.0, *N* = 34, *p* = .218).

### Pointing

As the goal of the present study was to investigate triadic communication, we ran additional analyses on the number of pointing gestures that participants produced in the experimenter’s line of sight (i.e., on the experimenter’s side or in the middle in-between sides). Contrary to the previous analyses, the following analyses focus on the target sides to which participants pointed instead of the sides where they were produced at.

**Fig 4:**
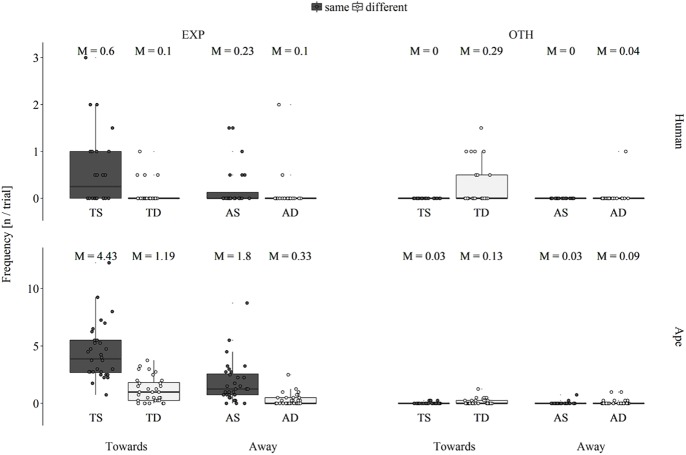
Box plot of the average number of pointing gestures per trial that human infants (upper row) and apes (lower row) directed towards the experimenter’s side (EXP: left column) and the other side (OTH: right column) across the factors orientation (Towards: two leftmost boxes in each plot, Away: two rightmost boxes in each plot) and location (red: experimenter and reward are on the same side, blue: experimenter and reward are on different sides) resulting in the four experimental conditions towards-same (TS), towards-different (TD), away-same (AS) and away-different (AD); M: condition mean value, the middle line of each box represent population median values (different from the M above each box); lower and upper hinges display first and third quartiles, respectively; whiskers extend up to 1.5 x interquartile range, small circles represent individual data points.

A Poisson GLMM indicated a significant overall effect of the test factors on the number of pointing gestures that participants directed towards the experimenter’s side (χ^2^ = 122.17, *df* = 7, *p* < .001; see Fig 4). This was due to participants pointing more frequently towards the experimenter’s side when the experimenter was oriented towards them (main effect of orientation: 1.04 ± 0.12, *p* < .001; see S7 Table) and when the reward was located on the same side (main effect of location: 1.40 ± 0.12, *p* < .001). Furthermore, apes pointed more frequently to the experimenter’s side than infants (main effect of species: 1.99 ± 0.28, *p* < .001).

As the GLMM for the number of pointing gestures that participants directed towards the other side failed to converge, we instead conducted within species pairwise comparisons across each of the two factors. Whereas infants and apes both pointed more frequently towards the other side when the reward was located there (Wilcoxon signed-ranks test; infants: *T*_*+*_ = 45.0, *N* = 9, *p* = .004; apes: *T*_*+*_ = 59.5, *N* = 11, *p* = .018), only infants exhibited a marginal trend to direct more pointing gestures towards the other side when the experimenter was attentive (Wilcoxon signed-ranks test; infants: *T*_*+*_ = 39.0, *N* = 9, *p* = .051; apes: *T*_*+*_ = 40.0, *N* = 11, *p* = .544).

## Discussion

Infants’ positioning as well as their production of auditory signals and visual gestures on the other side (which was out-of-sight for the experimenter in all conditions) was foremost driven by the reward location. Nevertheless, they generally exhibited a strong preference to request the reward at the experimenter’s side. Infants did not increase the frequency of their auditory signals to attract the attention of the experimenter when he was turned away. They did, however, exhibit a marginal trend to increase the frequency of their auditory signals when the experimenter was inattentive. A likely explanation for this finding might be that vocalizations, which constituted a major part of all auditory signals produced by infants (256 of 288), occurred frequently in conjunction with pointing and other visual gestures. Thus instead of employing them as attention getters, infants seemed to use their vocalizations that often were single word utterances like “hier” (here) and “da” (there) as a means to highlight and clarify the referent of their gesturing (accounting for 104 instances produced by 12 different infants). Nevertheless, the effect of the experimenter’s attention on infants’ auditory signals was less pronounced than for their visual gestures, which is in accordance with expectation.

While infants adapted their production of visual gestures on the experimenter’s side to the attentional state of the experimenter their visual gesturing remained unaffected by the location of the reward. This was due to infants also pointing in the experimenter’s line of sight when the reward was located on the other side. In contrast, apes preferentially approached the reward before pointing. Furthermore, infants adapted the direction of their pointing towards the experimenter’s and the other side in accordance with the location of the reward as well as the experimenter’s attentional state. Therefore one can rule out the possible explanation that this behaviour arose merely from the experimenter and the reward being in separate locations. One could argue that the infants were trying to command the experimenter to the reward location. However, this is not reflected by the single word utterances that frequently accompanied their pointing and that were never verbs or commands like “gib” (give) or “geh” (go), but only indicatives like “hier” (here) or “da” (there). This indicates that the infants’ pointing was triadic and constituted a communicative effort to inform the experimenter about the whereabouts of the reward.

This result is in line with a cognitive rich interpretation of infant pointing. Recent studies have not only found various prosocial motives for infant pointing such as providing helpful information [53], warning others [54], and sharing interest in objects and events [55], but findings also indicate referential intentions in infants’ pointing. For instance, in a study by Liszkowski et al. [56], in which infants were confronted with a recipient positively emoting to a wrong target, they persisted in their communicative effort until the recipient had correctly identified the event of interest. Shwe et al. [42] found infants to correct misunderstandings even after successfully obtaining the desired object, demonstrating that it plays a pivotal role for infants that the referent of their request is properly understood (see also [41]). Moreover, 12 month old infants seem capable to request absent objects by pointing to the empty place that previously held these objects [57].

Like the infants in our study, the apes’ positioning as well as their production of auditory signals and visual gestures on the other side was foremost driven by the reward location. Unlike infants, however, the reward location also influenced apes’ signaling on the experimenter’s side and they exhibited a predominant preference to gesture visually at the reward location. This result supports the findings of van der Goot et al. [46] and Leavens et al. [45]. Van der Goot et al. [46] showed that chimpanzees always approached the desired item as close as possible before signaling their request, whereas the 12 month old infants continued to point from the distance even when they could have retrieved the reward themselves. However, in their study, experimenter and reward were in the same location. Likewise, in the current study, the apes in the TS condition almost exclusively signaled on the side with the reward (and the experimenter). Leavens et al. [45] found no preference within their sample of chimpanzees to signal in close proximity to the reward in a setting where the experimenter and the reward were in different locations, while we found such a preference in the TD condition. This might be due to differences in the coding protocols across the two studies, since we coded and compared participants’ positioning in relation to the middle line that split the experimental area in half (with experimenter and reward being positioned equally distant from the middle). However, Leavens et al. [45] compared signals produced in close proximity of the reward to the summed signals produced close to the experimenter and anywhere else in-between those two extreme positions.

Like infants, apes did not increase the frequency of their auditory signals when the experimenter was turned away but instead exhibited a reversed trend to produce more auditory signals when the experimenter was attentive (turning significant when the ape sample is restricted to the two *Pan* species). This result is in line with others implying that apes do not primarily employ their auditory signals to attract the experimenter’s attention [23, 30–32]. Nevertheless, like infants, apes auditory signals were less affected by the experimenter’s orientation than their visual gestures.

Apes adapted the production of their visual gesturing on the experimenter’s side to the experimenter’s attentional state. However, unlike infants, the frequency of their visual gestures on the experimenter’s side was also strongly affected by the reward location. This was due to apes preferentially gesturing in proximity to the reward, even though this meant they would not be seen when the experimenter was positioned separate from the reward (note however that the two *Pan* species seemed to be less strongly affected by the reward location and more attuned to the experimenter’s orientation than the other ape species). Considering the target of the apes’ pointing—being positioned in the experimenter’s line of sight apes almost exclusively pointed towards the experimenter. The few pointing gestures directed to the other side occurred foremost from a position in-between sides and were solely driven by the reward location. Crucially, the number of pointing gestures directed towards the other side was not affected by the experimenter’s attentional state, suggesting that their pointing did not constitute a deliberate communicative effort to inform the experimenter about the whereabouts of the reward. The absence of statistical significance here might be due to a lack of sample size and thus statistical power, applying exactly the same tests to human infants we found that they pointed marginally more often to the other side when the experimenter was attentive, even though they were less in number (24 infants vs. 32 apes) and trials lasted 10s less for infants than for apes. Thus the finding that apes’ pointing to the other side remained unaffected by the experimenter’s attentional state seems to indicate that their pointing did not serve the goal to redirect the experimenter’s attention (either due to a motivational or cognitive lack).

Yet, the apes might have tried to indicate the reward location by other means. As Menzel [47] suggested inconspicuous body pointing as a possible mechanism for referential communication in chimpanzees, we also analysed the number of switches between sides. Indeed participants adapted their switches towards the other side in accordance to the reward location when the experimenter was attentive but not when he was facing away. However, although participants switched more often to the other side in the TD condition than in the TS condition, they did not switch there less often when the experimenter was inattentive (i.e., in the AD condition). Therefore it seems unlikely that the switches to the other side constituted a deliberate attempt to communicate about the reward location. However, referential communication via locomotion, as suggested by Menzel [47], might be more suited for longer distances. Thus, with the distance between the two locations being only about 1.5 m, our experimental set-up might have been inadequate to elicit such communicative behaviour. Finally, the apes might have employed more subtle cues that we did not identify as a means of (referential) communication, such as body postures directed towards the reward or gaze alternation between the reward and the experimenter.

Their pointing gestures, however, were not examples of triadic communication, since unlike infants, who pointed to direct the experimenter’s attention to the reward location, apes pointed imperatively to indicate the food they wanted to obtain. One potential explanation for this discrepancy between human infants and apes might be the differences in the experimental procedures; that the reward was hidden from the apes’ sight, whereas it was visible for infants. Yet, it seems unlikely that this prevented apes from engaging in triadic pointing. Apes clearly remembered the whereabouts of the hidden reward, and we consequently found a location effect on the production as well as the direction of their pointing. What differentiated their communication from that of infants, was rather the fact that the experimenter’s orientation had no effect on the apes’ pointing to the other side. Making the reward visible for apes would have, rather, reinforced the (already prepotent) influence of location and not the other way around. Another difference in the experimental procedure was that the infants were tested in a more playful setting than the apes, to keep the incentive to request for both species as strong as possible (as the apes were more motivated to request food than toys). The playful situation might have led infants to be more attuned to the attentional states of the experimenter (although *E1* only adopted a supporting role in the game). However, this is not supported by the GLMM analyses, which did not reveal a differential effect of orientation on infants and apes for any measure except for their pointing to the other side (post hoc tests for the only species x orientation interaction indicated no effects of orientation on the number of auditory gestures on the other side for both species). Thus it seems unlikely that apes’ failure to engage in triadic pointing was due to them being generally less attuned to the experimenter’s orientation than infants.

Instead, the results are in line with other studies casting doubt on whether apes point with referential intentions. Most notably in this regard, apes fail to comprehend declarative pointing (as referring to hidden food) in the object-choice task (e.g. [58,59]). This can be most likely explained by their cognitive restrictions and not the cooperative nature of the task, as shown by a recent study demonstrating the ape’s failure to use pointing gestures even in competitive situations [59]. Furthermore, although apes point imperatively, they seem to not comprehend imperative requests [58]. Hopkins et al. [60] recently challenged the latter claim by demonstrating that the chimpanzees successfully identified the one tube (amongst three) through which they should deliver a requested item based on the experimenter’s pointing cue. However, contrary to the task of Kirchhofer et al. [58], where participants were required to identify a distant referent which should be returned, in their task the pointing occurred in close proximity to the target tube (in a distance of 5–10 cm). Thus it might be that the apes in the study of Hopkins et al. [60] understood the human’s request, but were just using the tube closest to the human’s hand to barter the desired object.

As several studies have demonstrated, apes understand the actions of others in terms of intentional motives [61] and differentiate between attentive and inattentive individuals in communicative as well as competitive situations (e.g. [23,25]). Further, chimpanzees can keep track of what others have and have not seen in the past [62,63] and exhibit an implicit sensitivity to the false beliefs of others [64]. Thus, to some extent, apes assign somewhat abstract mental concepts to others to explain and predict their actions.

However, assigning mental states is not necessarily an all-or-nothing affair. Conceptualizing attention as a dynamic mental state that is modifiable and directable might be more challenging than understanding attention as something that can just be either present or absent (see [65] for a similar argument). The latter concept might be sufficiently flexible to predict whether others will react in various cooperative as well as competitive situations, and even enable inferences about whether others are knowledgeable (i.e., have attended) or ignorant (i.e., have not attended), as well as (implicit) knowledge about where others’ have last spotted an item (leading to true and false beliefs about entity locations; see [64]). However, if it comes to directing other’s attention to external objects and events such a concept seems to fail. This would also explain why apes primarily employ their attention-getting signals to draw attention to themselves, rather than using them to specifically draw attention to their visual gestures [66]. If given the chance to do so, they rather maneuver into the recipient’s line of sight [30]. Considering these findings, it seems not surprising that the only systematic observation of ape pointing in natural settings so far—a foot pointing gesture employed by female bonobos to solicit the recipient for genital-genital rubbing—was directed to the sender herself [33].

Nevertheless, apes are not genetically restricted to such a simplistic concept of attention, as home reared and language-trained apes have been reported to produce and comprehend declarative pointing (reviewed in [37]). While institutionalized apes facing physical restriction might spontaneously acquire the pointing gesture as a means to manipulate human caretakers to gain access to desirable but otherwise out-of-reach objects, the social environment of human infants is vastly different from the one that apes usually encounter in captivity. The socially enriched circumstances (e.g., by extended episodes of joint engagement initiated by human caretakers), under which human infants and language-trained apes are fostered, might contribute to an enhanced understanding of and a higher sensitivity to attentional (as well as other mental) states and thus lead to more sophisticated communicative strategies [45]. Furthermore, although the experimental conditions were designed as similar as possible, requesting from others is certainly a more commonplace experience for human infants and language-trained apes than standard-reared captive apes. Therefore, to investigate the performance of enculturated apes with a more rigorously controlled experimental paradigm to that of the current study might be an interesting venue for future research.

## Supporting information

S1 Dataset Raw data and summarized data for duration of stay, number of switches, auditory signals, visual and pointing gestures. (XLSX)

S1 Table GLMM analysis of number of switches to the experimenter’s side. (DOCX)

S2 Table GLMM analysis of number of switches to the other side. (DOCX)

S3 Table GLMM analysis of the number of auditory signals produced at the experimenter’s side. (DOCX)

S4 Table GLMM analysis of the number of auditory signals produced at the other side. (DOCX)

S5 Table GLMM analysis of the number of visual gestures produced at the experimenter’s side. (DOCX)

S6 Table GLMM analysis of the number of visual gestures produced at the other side. (DOCX)

S7 Table GLMM analysis of the number of pointing gestures directed to the experimenter’s side. (DOCX)

S1 Video TS condition. Towards-same condition; experimenter on the right side; reward on the right side.
(MP4)

S2 Video TD condition. Towards-different condition; experimenter on the left side; reward on the right side.
(MP4)

S3 Video AS condition. Away-same condition; experimenter on the left side; reward on the left side.
(MP4)

S4 Video AD condition. Away-different condition; experimenter on the right side; reward on the left side.
(MP4)
