# Supplementary material for: Prelinguistic human infants and great apes show different communicative strategies in a triadic request situation
Source: PLoS One. 2017 Apr 6;12(4):e0175227. doi: 10.1371/journal.pone.0175227 (PMC5383261; doi:10.1371/journal.pone.0175227)
Supplement: S1 Table — (DOCX) [file pone.0175227.s002.docx]

**S1 Table**

*GLMM analysis of number of switches to the experimenter’s side*

|  | | Model coefficients | | |  | Likelihood ratio tests | | |
| --- | --- | --- | --- | --- | --- | --- | --- | --- |
|  | | Estimate | SE | *p* |  | χ^2^ | *df* | *p* |
| Human, Great Apes | |  |  |  |  |  |  |  |
|  | Intercept | -1.40 | 0.27 | < .001 |  |  |  |  |
|  | Trial | -0.02 | 0.08 | .808 |  |  |  |  |
|  | Sex male | 0.04 | 0.23 | .847 |  |  |  |  |
|  | Species ape | 0.53 | 0.26 | .038 |  |  |  |  |
|  | Orientation towards | 0.32 | 0.15 | .031 |  |  |  |  |
|  | Location same | -0.67 | 0.25 | .008 |  |  |  |  |
|  | Species x Orientation |  |  |  |  | 0.09 | 1 | .771 |
|  | Species x Location |  |  |  |  | 0.10 | 1 | .752 |
|  | Orientation x Location | -0.61 | 0.26 | .021 |  | 5.43 | 1 | .020 |
|  | Species x Orientation x Location |  |  |  |  | 3.45 | 1 | .063 |
|  | **Test variables overall:** |  |  |  |  | 48.16 | 7 | < .001 |
| *Homo, Pan* | |  |  |  |  |  |  |  |
|  | Intercept | -1.30 | 0.26 | < .001 |  |  |  |  |
|  | Trial | 0.04 | 0.08 | .603 |  |  |  |  |
|  | Sex male | 0.00 | 0.23 | .992 |  |  |  |  |
|  | Species ape | 0.65 | 0.25 | .009 |  |  |  |  |
|  | Orientation towards | 0.30 | 0.16 | .061 |  |  |  |  |
|  | Location same | -0.49 | 0.25 | .045 |  |  |  |  |
|  | Species x Orientation |  |  |  |  | 0.18 | 1 | .674 |
|  | Species x Location |  |  |  |  | < 0.01 | 1 | .970 |
|  | Orientation x Location | -0.64 | 0.28 | .021 |  | 5.63 | 1 | .018 |
|  | Species x Orientation x Location |  |  |  |  | 3.82 | 1 | .051 |
|  | **Test variables overall:** |  |  |  |  | 40.81 | 7 | < .001 |
