# Supplementary material for: Prelinguistic human infants and great apes show different communicative strategies in a triadic request situation
Source: PLoS One. 2017 Apr 6;12(4):e0175227. doi: 10.1371/journal.pone.0175227 (PMC5383261; doi:10.1371/journal.pone.0175227)
Supplement: S2 Table — (DOCX) [file pone.0175227.s003.docx]

**S2 Table**

*GLMM analysis of number of switches to the other side*

|  | | Model coefficients | | |  | Likelihood ratio tests | | |
| --- | --- | --- | --- | --- | --- | --- | --- | --- |
|  | | Estimate | SE | *p* |  | χ^2^ | *df* | *p* |
| Human, Great Apes | |  |  |  |  |  |  |  |
|  | Intercept | -1.28 | 0.29 | < .001 |  |  |  |  |
|  | Trial | 0.05 | 0.09 | .593 |  |  |  |  |
|  | Sex male | 0.18 | 0.27 | .499 |  |  |  |  |
|  | Species ape | 0.01 | 0.28 | .959 |  |  |  |  |
|  | Orientation towards | 0.21 | 0.16 | .201 |  |  |  |  |
|  | Location same | 0.03 | 0.17 | .855 |  |  |  |  |
|  | Species x Orientation |  |  |  |  | 3.23 | 1 | .072 |
|  | Species x Location |  |  |  |  | 0.19 | 1 | .663 |
|  | Orientation x Location | -0.60 | 0.25 | .015 |  | 6.19 | 1 | .013 |
|  | Species x Orientation x Location |  |  |  |  | 2.09 | 1 | .148 |
|  | **Test variables overall:** |  |  |  |  | 15.93 | 7 | .026 |
| *Homo, Pan* | |  |  |  |  |  |  |  |
|  | Intercept | -1.14 | 0.28 | < .001 |  |  |  |  |
|  | Trial | 0.08 | 0.09 | .397 |  |  |  |  |
|  | Sex male | 0.08 | 0.26 | .753 |  |  |  |  |
|  | Species ape | 0.20 | 0.27 | .471 |  |  |  |  |
|  | Orientation towards | 0.20 | 0.17 | .248 |  |  |  |  |
|  | Location same | -0.03 | 0.18 | .862 |  |  |  |  |
|  | Species x Orientation |  |  |  |  | 3.07 | 1 | .080 |
|  | Species x Location |  |  |  |  | 0.34 | 1 | .561 |
|  | Orientation x Location | -0.54 | 0.26 | .039 |  | 4.57 | 1 | .032 |
|  | Species x Orientation x Location |  |  |  |  | 1.80 | 1 | .179 |
|  | **Test variables overall:** |  |  |  |  | 14.97 | 7 | .036 |
