# Supplementary material for: Prelinguistic human infants and great apes show different communicative strategies in a triadic request situation
Source: PLoS One. 2017 Apr 6;12(4):e0175227. doi: 10.1371/journal.pone.0175227 (PMC5383261; doi:10.1371/journal.pone.0175227)
Supplement: S3 Table — (DOCX) [file pone.0175227.s004.docx]

**S3 Table**

*GLMM analysis of the number of auditory signals produced at the experimenter’s side*

|  | | Model coefficients | | |  | Likelihood ratio tests | | |
| --- | --- | --- | --- | --- | --- | --- | --- | --- |
|  | | Estimate | SE | *p* |  | χ^2^ | *df* | *p* |
| Human, Great Apes | |  |  |  |  |  |  |  |
|  | Intercept | -0.86 | 0.32 | 0.006 |  |  |  |  |
|  | Trial | -0.19 | 0.12 | 0.106 |  |  |  |  |
|  | Sex male | 0.20 | 0.34 | 0.563 |  |  |  |  |
|  | Species ape | -2.67 | 0.40 | < .001 |  |  |  |  |
|  | Orientation towards | 0.34 | 0.20 | 0.098 |  |  |  |  |
|  | Location same | 0.04 | 0.24 | 0.862 |  |  |  |  |
|  | Species x Orientation |  |  |  |  | 0.28 | 1 | .594 |
|  | Species x Location | 1.51 | 0.39 | < .001 |  | 13.84 | 1 | < .001 |
|  | Orientation x Location |  |  |  |  | 0.20 | 1 | .651 |
|  | Species x Orientation x Location |  |  |  |  | 0.32 | 1 | .574 |
|  | **Test variables overall:** |  |  |  |  | 53.45 | 7 | < .001 |
| *Homo, Pan* | |  |  |  |  |  |  |  |
|  | Intercept | -0.87 | 0.33 | .009 |  |  |  |  |
|  | Trial | -0.14 | 0.12 | .255 |  |  |  |  |
|  | Sex male | 0.20 | 0.36 | .578 |  |  |  |  |
|  | Species ape | -2.63 | 0.44 | < .001 |  |  |  |  |
|  | Orientation towards | 0.45 | 0.20 | .029 |  |  |  |  |
|  | Location same | 0.04 | 0.26 | .884 |  |  |  |  |
|  | Species x Orientation |  |  |  |  | < 0.01 | 1 | .956 |
|  | Species x Location | 1.55 | 0.44 | < .001 |  | 11.78 | 1 | .001 |
|  | Orientation x Location |  |  |  |  | 0.07 | 1 | .789 |
|  | Species x Orientation x Location |  |  |  |  | 0.12 | 1 | .732 |
|  | **Test variables overall:** |  |  |  |  | 45.38 | 7 | < .001 |
