# Supplementary material for: Prelinguistic human infants and great apes show different communicative strategies in a triadic request situation
Source: PLoS One. 2017 Apr 6;12(4):e0175227. doi: 10.1371/journal.pone.0175227 (PMC5383261; doi:10.1371/journal.pone.0175227)
Supplement: S4 Table — (DOCX) [file pone.0175227.s005.docx]

**S4 Table**

*GLMM analysis of the number of auditory signals produced at the other side*

|  | | Model coefficients | | |  | Likelihood ratio tests | | |
| --- | --- | --- | --- | --- | --- | --- | --- | --- |
|  | | Estimate | SE | *p* |  | χ^2^ | *df* | *P* |
| Human, Great Apes | |  |  |  |  |  |  |  |
|  | Intercept | -1.1 | 0.41 | .007 |  |  |  |  |
|  | Trial | 0.08 | 0.17 | .632 |  |  |  |  |
|  | Sex male | 0.09 | 0.40 | .815 |  |  |  |  |
|  | Species ape | -1.33 | 0.46 | .004 |  |  |  |  |
|  | Orientation towards | 0.25 | 0.28 | .376 |  |  |  |  |
|  | Location same | -4.09 | 1.95 | .036 |  |  |  |  |
|  | Species x Orientation | -0.98 | 0.46 | .033 |  | 3.89 | 1 | .048 |
|  | Species x Location |  |  |  |  | 3.77 | 1 | .052 |
|  | Orientation x Location |  |  |  |  | 0.11 | 1 | .735 |
|  | Species x Orientation x Location |  |  |  |  | 2.08 | 1 | .149 |
|  | **Test variables overall:** |  |  |  |  | 64.68 | 7 | < .001 |
| *Homo, Pan* | |  |  |  |  |  |  |  |
|  | Intercept | -1.04 | 0.42 | .013 |  |  |  |  |
|  | Trial | 0.05 | 0.19 | .786 |  |  |  |  |
|  | Sex male | 0.12 | 0.42 | .773 |  |  |  |  |
|  | Species ape | -1.61 | 0.45 | < .001 |  |  |  |  |
|  | Orientation towards | -0.04 | 0.27 | .886 |  |  |  |  |
|  | Location same | -3.23 | 1.52 | .033 |  |  |  |  |
|  | Species x Orientation |  |  |  |  | 2.51 | 1 | .113 |
|  | Species x Location |  |  |  |  | 3.11 | 1 | .078 |
|  | Orientation x Location |  |  |  |  | 0.08 | 1 | 771 |
|  | Species x Orientation x Location |  |  |  |  | 2.33 | 1 | .127 |
|  | **Test variables overall:** |  |  |  |  | 50.23 | 7 | < .001 |
