# Supplementary material for: Prelinguistic human infants and great apes show different communicative strategies in a triadic request situation
Source: PLoS One. 2017 Apr 6;12(4):e0175227. doi: 10.1371/journal.pone.0175227 (PMC5383261; doi:10.1371/journal.pone.0175227)
Supplement: S5 Table — (DOCX) [file pone.0175227.s006.docx]

**S5 Table**

*GLMM analysis of the number of visual gestures produced at the experimenter’s side*

|  | | Model coefficients | | |  | Likelihood ratio tests | | |
| --- | --- | --- | --- | --- | --- | --- | --- | --- |
|  | | Estimate | SE | *p* |  | χ^2^ | *df* | *p* |
| Human, Great Apes | |  |  |  |  |  |  |  |
|  | Intercept | -1.62 | 0.3 | < .001 |  |  |  |  |
|  | Trial | 0.00 | 0.05 | .960 |  |  |  |  |
|  | Sex male | -0.29 | 0.27 | .297 |  |  |  |  |
|  | Species ape | 0.27 | 0.31 | .381 |  |  |  |  |
|  | Orientation towards | 1.07 | 0.13 | < .001 |  |  |  |  |
|  | Location same | 0.27 | 0.27 | .316 |  |  |  |  |
|  | Species x Orientation |  |  |  |  | 0.72 | 1 | .397 |
|  | Species x Location | 1.39 | 0.31 | < .001 |  | 20.39 | 1 | < .001 |
|  | Orientation x Location |  |  |  |  | 0.46 | 1 | .499 |
|  | Species x Orientation x Location |  |  |  |  | 0.41 | 1 | .520 |
|  | **Test variables overall:** |  |  |  |  | 103.85 | 7 | < .001 |
| *Homo, Pan* | |  |  |  |  |  |  |  |
|  | Intercept | -1.72 | 0.30 | < .001 |  |  |  |  |
|  | Trial | -0.04 | 0.07 | .521 |  |  |  |  |
|  | Sex male | -0.25 | 0.28 | .359 |  |  |  |  |
|  | Species ape | 0.41 | 0.31 | .181 |  |  |  |  |
|  | Orientation towards | 1.17 | 0.14 | < .001 |  |  |  |  |
|  | Location same | 0.26 | 0.27 | .346 |  |  |  |  |
|  | Species x Orientation |  |  |  |  | 1.88 | 1 | .170 |
|  | Species x Location | 1.36 | 0.33 | < .001 |  | 18.17 | 1 | < .001 |
|  | Orientation x Location |  |  |  |  | 1.62 | 1 | .203 |
|  | Species x Orientation x Location |  |  |  |  | 0.13 | 1 | .723 |
|  | **Test variables overall:** |  |  |  |  | 84.31 | 7 | < .001 |
