# Supplementary material for: Prelinguistic human infants and great apes show different communicative strategies in a triadic request situation
Source: PLoS One. 2017 Apr 6;12(4):e0175227. doi: 10.1371/journal.pone.0175227 (PMC5383261; doi:10.1371/journal.pone.0175227)
Supplement: S6 Table — (DOCX) [file pone.0175227.s007.docx]

**S6 Table**

*GLMM analysis of the number of visual gestures produced at the other side*

|  | | Model coefficients | | |  | Likelihood ratio tests | | |
| --- | --- | --- | --- | --- | --- | --- | --- | --- |
|  | | Estimate | SE | *p* |  | χ^2^ | *df* | *p* |
| Human, Great Apes | |  |  |  |  |  |  |  |
|  | Intercept | -1.78 | 0.35 | < .001 |  |  |  |  |
|  | Trial | -0.05 | 0.09 | .572 |  |  |  |  |
|  | Sex male | -0.26 | 0.30 | 0.392 |  |  |  |  |
|  | Species ape | 1.83 | 0.35 | < .001 |  |  |  |  |
|  | Orientation towards | 0.15 | 0.13 | .253 |  |  |  |  |
|  | Location same | -3.17 | 0.65 | < .001 |  |  |  |  |
|  | Species x Orientation |  |  |  |  | 0.11 | 1 | .744 |
|  | Species x Location |  |  |  |  | 1.23 | 1 | .267 |
|  | Orientation x Location | -1.89 | 0.57 | .001 |  | 14.41 | 1 | < .001 |
|  | Species x Orientation x Location |  |  |  |  | 0.96 | 1 | .328 |
|  | **Test variables overall:** |  |  |  |  | 124.97 | 7 | < .001 |
| *Homo, Pan* | |  |  |  |  |  |  |  |
|  | Intercept | -1.89 | 0.37 | < .001 |  |  |  |  |
|  | Trial | -0.16 | 0.1 | .109 |  |  |  |  |
|  | Sex male | -0.22 | 0.33 | .501 |  |  |  |  |
|  | Species ape | 2.02 | 0.37 | < .001 |  |  |  |  |
|  | Orientation towards | 0.20 | 0.15 | .182 |  |  |  |  |
|  | Location same | -3.29 | 0.75 | < .001 |  |  |  |  |
|  | Species x Orientation |  |  |  |  | 0.23 | 1 | .635 |
|  | Species x Location |  |  |  |  | 1.70 | 1 | .192 |
|  | Orientation x Location | -1.49 | 0.61 | .014 |  | 6.66 | 1 | .010 |
|  | Species x Orientation x Location |  |  |  |  | 0.46 | 1 | .499 |
|  | **Test variables overall:** |  |  |  |  | 105.39 | 7 | < .001 |
