# Supplementary material for: Prelinguistic human infants and great apes show different communicative strategies in a triadic request situation
Source: PLoS One. 2017 Apr 6;12(4):e0175227. doi: 10.1371/journal.pone.0175227 (PMC5383261; doi:10.1371/journal.pone.0175227)
Supplement: S7 Table — (DOCX) [file pone.0175227.s008.docx]

**S7 Table**

*GLMM analysis of the number of pointing gestures directed to the experimenter’s side*

|  | | Model coefficients | | |  | Likelihood ratio tests | | |
| --- | --- | --- | --- | --- | --- | --- | --- | --- |
|  | | Estimate | SE | *p* |  | χ^2^ | *df* | *p* |
| Human, Great Apes | |  |  |  |  |  |  |  |
|  | Intercept | -3.02 | 0.30 | < .001 |  |  |  |  |
|  | Trial | -0.01 | 0.05 | .781 |  |  |  |  |
|  | Sex male | -0.41 | 0.25 | .096 |  |  |  |  |
|  | Species ape | 1.99 | 0.28 | < .001 |  |  |  |  |
|  | Orientation towards | 1.04 | 0.12 | < .001 |  |  |  |  |
|  | Location same | 1.40 | 0.12 | < .001 |  |  |  |  |
|  | Species x Orientation |  |  |  |  | 1.07 | 1 | .300 |
|  | Species x Location |  |  |  |  | 0.20 | 1 | .656 |
|  | Orientation x Location |  |  |  |  | 2.01 | 1 | .156 |
|  | Species x Orientation x Location |  |  |  |  | 0.24 | 1 | .625 |
|  | **Test variables overall:** |  |  |  |  | 122.17 | 7 | < .001 |
| *Homo, Pan* | |  |  |  |  |  |  |  |
|  | Intercept | -3.15 | 0.32 | < .001 |  |  |  |  |
|  | Trial | -0.05 | 0.07 | .491 |  |  |  |  |
|  | Sex male | -0.48 | 0.27 | .079 |  |  |  |  |
|  | Species ape | 2.13 | 0.30 | < .001 |  |  |  |  |
|  | Orientation towards | 1.14 | 0.14 | < .001 |  |  |  |  |
|  | Location same | 1.45 | 0.14 | < .001 |  |  |  |  |
|  | Species x Orientation |  |  |  |  | 1.91 | 1 | .166 |
|  | Species x Location |  |  |  |  | 0.37 | 1 | .544 |
|  | Orientation x Location |  |  |  |  | 1.22 | 1 | .269 |
|  | Species x Orientation x Location |  |  |  |  | 0.20 | 1 | .652 |
|  | **Test variables overall:** |  |  |  |  | 98.25 | 7 | < .001 |
